# The ß-importin KAP8 (Pse1/Kap121) is required for nuclear import of the cellulase transcriptional regulator XYR1, asexual sporulation and stress resistance in Trichoderma reesei

**DOI:** 10.1111/mmi.12944

**Published:** 2015-03-04

**Authors:** Sara Ghassemi, Alexander Lichius, Fréderique Bidard, Sophie Lemoine, Marie-Noëlle Rossignol, Silvia Herold, Verena Seidl-Seiboth, Bernhard Seiboth, Eduardo A Espeso, Antoine Margeot, Christian P Kubicek

**Affiliations:** 1 Research Division Biotechnology and Microbiology, Institute of Chemical EngineeringTU Wien, Vienna, 1060, Austria; 2 IFP Energies nouvelles1-4 avenue de Bois-Préau, 92852, Rueil-Malmaison, France; 3 Ecole Normale Supérieure, Institut de Biologie de l'ENSIBENS, Plateforme Génomique, Paris, F-75005, France; 4 ACIB GmbH, c/o Institute of Chemical EngineeringTU Wien, Vienna, 1060, Austria; 5 Department of Cellular and Molecular Biology, Centro de Investigaciones Biológicas, Consejo Superior de Investigaciones CientíficasMadrid, Spain

## Abstract

The ascomycete *Trichoderma reesei* is an industrial producer of cellulolytic and hemicellulolytic enzymes, and serves as a prime model for their genetic regulation. Most of its (hemi-)cellulolytic enzymes are obligatorily dependent on the transcriptional activator XYR1. Here, we investigated the nucleo-cytoplasmic shuttling mechanism that transports XYR1 across the nuclear pore complex. We identified 14 karyopherins in *T. reesei*, of which eight were predicted to be involved in nuclear import, and produced single gene-deletion mutants of all. We found KAP8, an ortholog of *Aspergillus nidulans* KapI, and *Saccharomyces cerevisiae* Kap121/Pse1, to be essential for nuclear recruitment of GFP-XYR1 and cellulase gene expression. Transformation with the native gene rescued this effect. Transcriptomic analyses of Δ*kap8* revealed that under cellulase-inducing conditions 42 CAZymes, including all cellulases and hemicellulases known to be under XYR1 control, were significantly down-regulated. Δ*kap8* strains were capable of forming fertile fruiting bodies but exhibited strongly reduced conidiation both in light and darkness, and showed enhanced sensitivity towards abiotic stress, including high osmotic pressure, low pH and high temperature. Together, these data underscore the significance of nuclear import of XYR1 in cellulase and hemicellulase gene regulation in *T. reesei*, and identify KAP8 as the major karyopherin required for this process.

## Introduction

*Trichoderma reesei* is today used for the industrial production of plant cell wall degrading enzymes applied in the pulp and paper, food and textile industries, as well as for the conversion of plant biomass materials into bioethanol or platform chemicals (Kubicek, 2012). Consequently, *T. reesei* has become the key experimental model system for the genetic and metabolic regulation of production of cellulases and hemicellulases (Seiboth *et al*., 2012; Amore *et al*., 2013; Kubicek, 2013; Tani *et al*., 2014).

Cellulase gene expression is adaptive and in *T. reesei* regulated by the action of at least four transcriptional activators (XYR1, ACE2, ACE3 and the HAP2/3/5 complex) and two repressors (ACE1 and the carbon catabolite repressor CRE1) (for review, see Seiboth *et al*., 2012; Amore *et al*., 2013; Häkkinen *et al*., 2014; Tani *et al*., 2014). XYR1, a Zn(2)Cys(6)-type transcriptional activator that binds to a 5′-GGCW_4_-3′ motif, plays a key role because its deletion completely eliminates the induction of cellulases and hemicellulases by all known inducers, including cellulose, lactose, sophorose, xylan and xylose (Stricker *et al*., 2008). XYR1 is constitutively formed at a very low basal level and becomes up-regulated and shuttled into the nucleus in an auto-regulatory feedback manner after addition of an inducer such as lactose or sophorose (Lichius *et al*., 2014).

The nucleus is surrounded by a double membrane, called the nuclear envelope (NE). The nucleoplasm and cytoplasm communicate through multiprotein complexes inserted in the NE called nuclear pores. Molecules smaller than 30 kDa are able to move passively through nuclear pores (Görlich and Kutay, 1999), whereas larger molecules require active transport with the help of specific nuclear carriers. The majority of these transporters belong to the karyopherin-β superfamily (KAPs) classified into importins and exportins (Mosammaparast and Pemberton, 2004), which serve as receptors for the import and/or export of diverse cargo molecules such as proteins and tRNAs (Görlich and Kutay, 1999).

The karyopherin-ß family is typically defined by a 150-amino acid region required for binding to the small GTPase Ran (Görlich, 1998; Pemberton *et al*., 1998). The archetype of their canonical nuclear targeting signal is the SV40 large T antigen nuclear localization signal that is rich in basic amino acids (Kalderon *et al*., 1984; Lanford and Butel, 1985). It is recognized by importin α, to which it binds thus forming an adaptor (Lange *et al*., 2007), which is subsequently bound by one of the importin ß proteins to form an import complex. This cargo-importin α-importin ß import complex then docks at the nuclear pore and translocates across the NE. Subsequently, the import complex is dissociated by binding of the small GTPase RanGTP to the importin ß, which releases the cargo into the nucleoplasm (Görlich and Kutay, 1999). The karyopherins are usually recycled into the cytoplasm for additional rounds of cargo import (Gilchrist *et al*., 2002).

With regard to Zn(2)Cys(6) cluster transcription factors, no general strategy for their import has been detected thus far (MacPherson *et al*., 2014). The best known member of fungal binuclear zinc cluster transcription factors, Gal4 of *Saccharomyces cerevisiae*, interacts directly with the ß-importin receptor Rsl1/Kap95 complex, as well as with another importin, Nmd5 (Chan and Jans, 1999). Another *S. cerevisiae* Zn(2)Cys(6) protein the ABC transporter regulator Pdr1 uses the Pse1/Kap121 complex (Delahodde *et al*., 2001).

In filamentous fungi, karyopherins have so far only been identified and studied in *Aspergillus nidulans* (Todd *et al*., 2005; Osmani *et al*., 2006; Araújo-Bazán *et al*., 2009; Etxebeste *et al*., 2009; Markina-Iñarrairaegui *et al*., 2011) and *Neurospora crassa* (Takeda *et al*., 2013). Yet information as to their involvement in nuclear transport of Zn(2)Cys(6) cluster transcription factors is still sparse: the regulator of the *A. nidulans* ethanol regulon, AlcR, for instance requires three importin-related proteins, Kap104, Sxm1 and Nmd5 (Nikolaev *et al*., 2003), whereas the rate-limiting step of nitrate regulation in the same fungus has been shown to be KapK-dependent (= CRM1/exportin 1) export of the pathway-specific regulator NirA (Bernreiter *et al*., 2007).

In order to identify the ß-importin(s) that transport *T. reesei* XYR1 into the nucleus under cellulase and hemicellulase inducing conditions, we investigated the functional role of all importin ß proteins encoded in the *T. reesei* genome. Using a systematic gene deletion approach, we identified KAP8, the ortholog of Pse1/Kap121from *S. cerevisiae* and *A. nidulans* KapI, respectively, to be required for XYR1 import into the nucleus, and demonstrate that this step is essential for cellulase and hemicellulase gene expression. In addition, we show that KAP8 is involved in asexual sporulation and response to abiotic stress.

## Results

### Identification of the karyopherin-ß superfamily in *T*. *reesei*

We searched the *T. reesei* genome (http://genome.jgi-psf.org/Trire2/Trire2.home.html) for potential orthologs of the 17 nuclear transporters previously identified in *A. nidulans* (Mans *et al*., 2004; Espeso and Osmani, 2008; Markina-Iñarrairaegui *et al*., 2011). Seventeen loci coding for nuclear transporters were found, of which 14 encoded proteins belonging to the karyopherin-β superfamily. Each of them showed a high similarity with only a single *A. nidulans* Kap protein (e-values between e-170 and 0). In agreement with Pyrenomycete nomenclature, these genes and proteins were designated as *kap/*KAP plus locus numbers from 1 to 14 (Table 1). They comprised four potential exportins (KAP11 = *A. nidulans* KapK^Crm1^, KAP13 = KapN, KAP5 = KapE^Cse1^, KAP12 = KapM^Los1^), nine ß-importins (KAP2 = KapB^Kap95^, KAP3 = KapC^Kap104^, KAP4 = KapD^Nmd5^, KAP6 = KapG^Kap114^, KAP7 = KapH^Kap120^, KAP8 = KapI^Pse1^, KAP9 = KapJ^Kap123^, KAP10 = KapL and the mRNA transporter KAP14 = KapF^Mtr10^; superscripts refer to the name of the corresponding yeast orthologs, if any) and the importin-α homologue KAP1 = KapA^Srp1^. Additional *in silico* searches using Pfam domains related to this superfamily of proteins did not add more candidates to our predictions (data not shown).

**Table 1:** Nuclear transporters in *Trichoderma reesei*

	Protein name			
Trire2:	*T. reesei*	*A. nidulans*	Protein type	Gene function in *A. nidulans*
21117	KAP1	KapA	Importin alpha	Essential
74318	KAP2	KapB	Importin beta	Essential
80398	KAP3	KapC	Importin beta	Nonessential
76859	KAP4	KapD	Importin beta	Nonessential
73525	KAP5	KapE	Importin beta	Essential
104161	KAP6	KapG	Importin beta	Nonessential
64009	KAP7	KapH	Importin beta	Nonessential
78158	KAP8	KapI	Importin beta	Nonessential
23193	KAP9	KapJ	Importin beta	Nonessential
77404	KAP10	KapL	Importin beta	Nonessential
62120	KAP11	KapK	Exportin	Essential
3892	KAP12	KapM	Exportin	Nonessential
5991	KAP13	KapN	Exportin	Nonessential
120152	KAP14	KapF	mRNA transporter Mtr10	Essential

### *Functional analysis of the* T. reesei *ß-importins reveals that kap8 is essential for cellulase gene expression in* T. reesei

To potentially identify the ß-importin of *T. reesei* that is responsible for XYR1 import, we generated single-knockout mutants for each locus by means of a precise gene replacement procedure (see *Experimental procedures*). The deletion of most of these importins produced viable homokaryotic colonies, demonstrating that these genes are not essential. Homokaryotic transformants could not be obtained, however, when deleting *kap1*, *kap2* and *kap5*, suggesting that these are essential genes in *T. reesei*.

**Figure 1:**
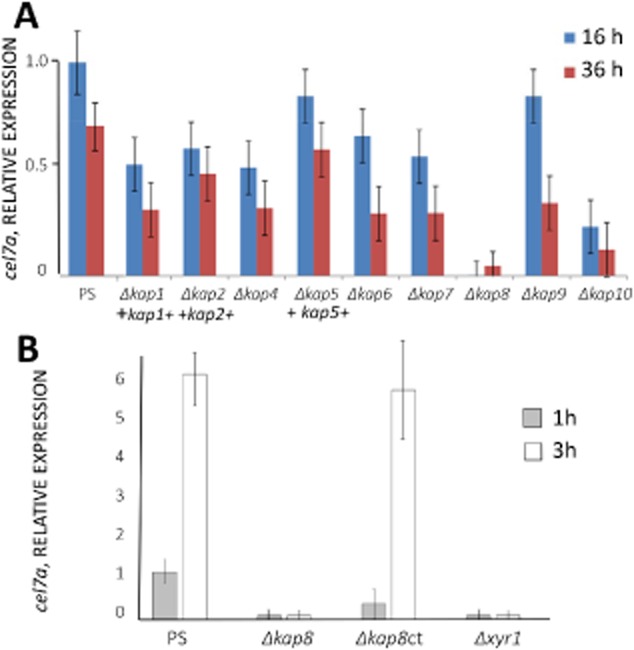
Expression of the *T. reesei* cellobiohydrolase *cel7A* gene during (A) growth on lactose for 16 h (blue bars) and 36 h (red bars) in nine ß-importin knockout strains. Values are given as ‘relative gene expression’, which is the ratio of expression of *cel7A* to that of the housekeeping gene *tef1*, normalized to the same ratio obtained with the parental strain *T. reesei* QM9414 after 16 h on lactose.B. Expression of *cel7A* in *T. reesei* parental strain (PS), *Δkap8* and its complemented transformant Δ*kap8ct*, and Δ*xyr1* after 24 h preculture on 1% (w/v) glycerol and subsequent transfer to 1.4 mM sophorose for 1 h (grey bars) and 3 h (white bars) respectively. Relative expression levels are defined as above, but the ratio obtained for the retransformant at t = 0 was used for normalization. Error bars indicate the standard deviation from *n* ≥ 3 biological replicates.

We then cultivated the knockout strains, the heterokaryon strains of *kap1*, *kap2* and *kap5*, and the parental strain of *T. reesei* on lactose to induce cellulase gene expression, and analyzed the level of the *cel7A* transcript (which encodes the major cellulase, *cbh1 = cel7A*), a product of nuclear function of XYR1. Lactose, rather than cellulose, was chosen for these experiments because lactose utilization – in contrast to cellulose – is independent of the action of secreted cellulases (Seiboth *et al*., 2007). The data are shown in Fig. 1A: the knockout strains exhibited somewhat reduced cel7A expression, but most strains (including the heterokaryon strains) showed expression levels of > 50% of the parental strain. Two exceptions were of note: *Δkap10*, which showed less than 30% of cel7A expression, and the Student's *t*-test determined this as significant (*P* = 0.00279 for 16 h, and *P* = 0.00998 for 36 h). In addition, *Δkap8* produced almost no cbh1/cel7A transcript. Student's *t*-test confirmed the significance of this result (*P* = 0.0000023 and 0.00029 for 16 and 36 h respectively). Subsequent transformation of the *Δkap8* strain with the wild-type *kap8* allele fully restored *cel7A* expression to the level shown by the parent strain, indicating that the above noted reduction was indeed dependent on *kap8* function.

In order to support these findings by an independent experiment, we pre-cultured the *Δkap8* strain, its complemented transformant strain and the parent strain on glycerol as carbon source, and then transferred the biomass into fresh medium containing 1.4 mM sophorose, another very potent cellulase inducer (Sternberg and Mandels, 1979), as sole carbon source. As shown in Fig. 1B, expression of *cbh1* in the *Δkap8* strain was only 2% of that shown by the complemented transformant strain. The *T. reesei Δxyr1* strain displayed similar *cel7A* expression levels as the *Δkap8* strain (Fig. 1B). We therefore concluded that *kap8* is essential for cellulase gene expression and – while it cannot be ruled out that other importins can also transport XYR1 into the nucleus – is of major importance for the function of XYR1.

### KAP8 is essential for nuclear import of XYR1 in *T. reesei*

**Figure 2:**
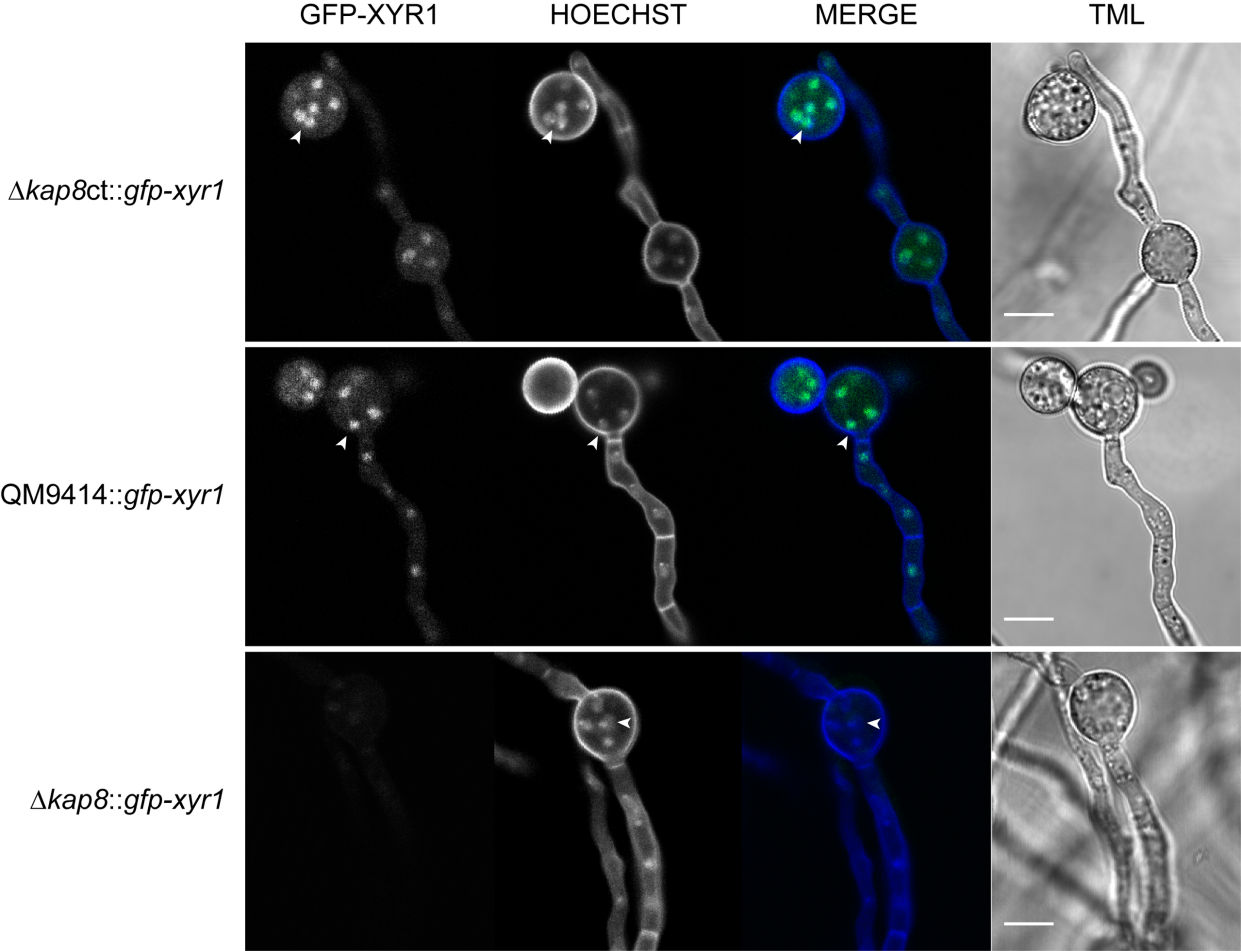
Nuclear recruitment of GFP-XYR1 was not observed in the Δ*kap8* strain; however, it was restored in the Δ*kap8* complemented transformat *(Δkap8*ct). In both cases, expression of *gfp-xyr1* was under control of the native *xyr1* promoter. On the left, fluorescent images are shown; on the right, the corresponding transmitted light images. Arrowheads indicate nuclei containing GFP-XYR1 and/or Hoechst dye. Scale bars, 10 μm.

In order to test whether KAP8 is indeed essential for XYR1 uptake into the nucleus, we expressed a GFP-XYR1 fusion protein in *T. reesei Δkap8* and its complemented transformant and monitored its subcellular localization under inducing conditions. As reported previously (Lichius *et al*., 2014), nuclear import of XYR1 in response to a cellulase inducing signal is essential to activate *xyr1* expression in an auto-regulatory manner and hence to produce sufficient amounts of XYR1 to elicit high-level cellulase and hemicellulase gene expression. As shown in Fig. 2, fluorescence microscopy demonstrated that the parent strain and the *kap8* complemented transformant imported XYR1 into the nuclei, whereas *Δkap8* did not. These data show that nuclear import of XYR1 is dependent on KAP8 function.

### KAP8 *is essential for the expression of the sophorose-induced plant cell wall degrading enzymes in* T. reesei

The results described above provided the first evidence that KAP8 is essential for nuclear import of XYR1 and is indirectly important for the formation of the cellulase CEL7A. The expression of all cellulase genes of *T. reesei* is known to be co-regulated by XYR1 (Kubicek, 2013). A similar effect of *kap8* on the expression of the other cellulase genes can be safely assumed. However, XYR1 also regulates the expression of several hemicellulases (Stricker *et al*., 2008; Seiboth *et al*., 2012; Amore *et al*., 2013), and its full regulon in *T. reesei* has not yet been identified. We investigated the global change in gene transcription in the *Δkap8* strain and its complemented transformant upon induction with 1.4 mM sophorose after 3 h. Transcriptomic analysis was based on the mean values from three experimental replicates.

**Figure 3:**
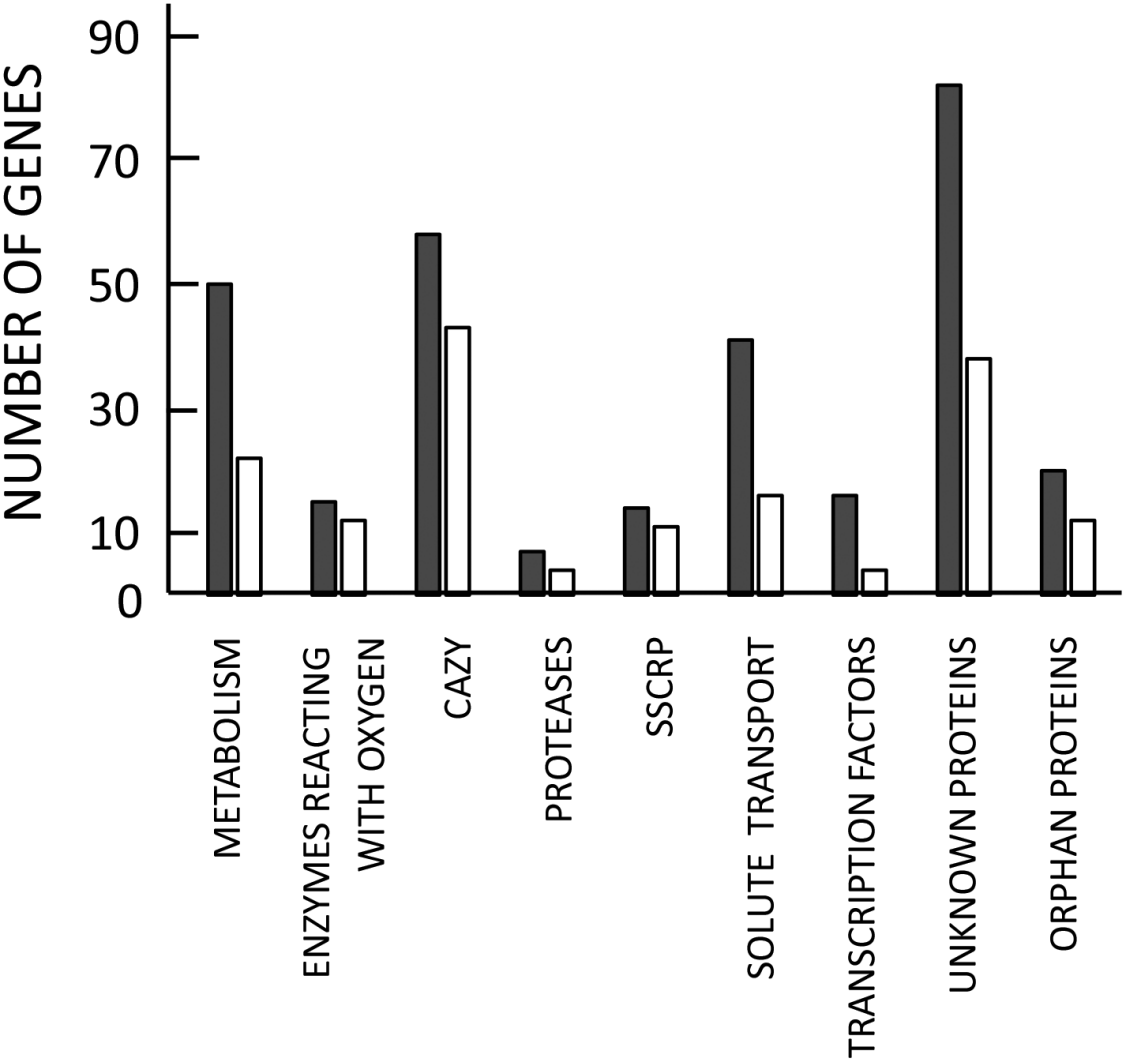
Number of genes, grouped according to functional similarity, that were induced by sophorose at least fourfold (grey bars) and genes whose sophorose induction was significantly lower (> 5-fold) in the *Δkap8* strain compared with the complemented transformant Δ*kap8ct* (white bars). The grouping shown comprised 81.7% of all sophorose-inducible genes of strain *Δkap8*ct as documented in Supporting Information Table S4. Gene groups and abbreviations: METABOLISM, genes involved in intracellular catabolism, anabolism and secondary metabolism; CAZY, all extracellular glycosyl hydrolases, carbohydrate esterases and accessory enzymes; ENZYMES REACTING WITH OXYGEN, oxidases, cytochrome P450- and FAD monooxygenases, dioxygenases; SSCRP, small, secreted, cysteine-rich proteins including hydrophobins and ceratoplatanins; SOLUTE TRANSPORT, members of the major facilitator superfamily; amino acid transport; ion transport; UNKNOWN PROTEINS, proteins that have orthologs in other species, but for which no function has as yet been described; and ORPHAN PROTEINS, proteins that have orthologs, if at all, only in other *Trichoderma* species, and for which also no function is as yet known.

We detected 351 genes (including 57 CAZymes) that were at least fourfold higher expressed on sophorose compared with the noninduced control (24 h glycerol preculture; Supporting Information Table S4). One hundred forty-six from them (including 44 CAZymes) were > 10-fold induced. Apart of CAZymes, genes encoding unknown conserved proteins, metabolic enzymes and membrane proteins that function in solute uptake were most abundant (Fig. 3). The rest of the genes identified (4–5% of the transcriptome) consisted of genes encoding enzymes which react with molecular oxygen, genes encoding small secreted cysteine-rich proteins (SSCRPs) and transcription factors (two C2H2-type, each one of BZIP-, myb- and MYND/zinc finger type, and 11 Zn(2)Cys(6) zinc cluster proteins; Supporting Information Table S4). The latter also included the already known regulatory genes *xyr1*, *clr2* (Trire2:26163, which encodes the ortholog of the *N. crassa* and *A. nidulans* cellulase regulator CLR-2/ClrB; Coradetti *et al*., 2012; Häkkinen *et al*., 2014) and *ace3* (Häkkinen *et al*., 2014).

In the *Δkap8* strain, 195 of the 351 sophorose-induced genes were more than fivefold down-regulated compared with the parental control and exhibited almost no expression irrespective of the presence or absence of sophorose (Supporting Information Table S5). They included 42 of the 57 CAZyme-encoding genes (73.7%; Table 2) and comprised all cellulase and hemicellulase genes that were known or believed to be under control of XYR1, as well as many other genes for which XYR1 control has not yet been detected, such as α–D-galactosidases, α-D-fucosidases, α-D-mannosidases, AA9 polysaccharide monooxygenases and polygalacturonases. Besides CAZymes, also several members of other gene groups listed in Fig. 3 exhibited significantly reduced expression in *Δkap8* strain, which was most pronounced with genes encoding proteases and SSCRPs (Fig. 3). The induction of *xyr1*, which itself is also induced by sophorose (*vide supra*), was only somewhat reduced (39% after 3 h of induction respectively; *P* < 0.001).

**Table 2:** GH, CE and AAs of *T. reesei* that are induced by sophorose and impaired in their expression in *Δkap8*

		RPKM			
		*Δkap8*		*Δkap8*RT	
Trire2:	Annotation	soph	glyc	Soph	*Δkap8*ct/*Δkap8*^a^ (-fold)
73643	AA9 polysaccharide monooxygenase CEL61a	548.6	9.5	76 189.2	138.9
65215	CE4 imidase	200.5	20.1	3 640.5	18.2
73632	CE5 acetyl xylan esterase AXE1	7.0	1.2	2 846.3	408.8
73638	CIP1	108.6	2.4	83 482.6	768.7
57179	GH105/GH88 glycosyl hydrolase	4.2	0.0	213.7	51.2
74223	GH11 endo-ß-1,4-xylanase XYN1	9.1	0.0	314.4	34.7
112392	GH11 endo-ß-1,4-xylanase XYN5	4.2	0.0	289.8	69.4
123232	GH12 endo-ß-1,4-glucanase	1.4	0.0	180.8	129.9
65162	GH18 endo-N-acetyl-ß-D-glucosaminidase Endo T	100.3	20.1	2 195.8	21.9
5836	GH2 β-mannosidase	72.4	0.0	3 192.5	44.1
102909	GH2 ß-glycosidase	53.6	66.4	294.9	5.5
55999	GH27 α-galactosidase	399.6	15.4	2 259.5	5.7
72632	GH27 α-galactosidase AGL1	14.6	1.2	160.3	11.0
72704	GH27 α-galactosidase AGL3	192.9	17.8	4 165.6	21.6
112140	GH28 exo-polygalacturonase PGX1	7.0	0.0	706.9	101.5
122780	GH28 exo-rhamnogalacturonase RGX1	669.1	39.1	50 745.5	75.8
108671	GH3β-glucosidase/glucan1,4-β-glucosidase BGL3f	7.7	0.0	167.5	21.9
76672	GH3 β-glucosidase BGL1/CEL3a	2.8	0.0	132.6	47.6
46816	GH3 β-glucosidase CEL3d	146.9	26.1	1865.0	12.7
121127	GH3 β-xylosidase BXL1	271.5	1.2	23 568.4	86.8
110894	GH30 endo-β-1 6-galactanase	104.4	58.1	534.3	5.1
69276	GH30 endo-β-1,4-xylanase	2.1	0.0	1 762.2	843.7
111849	GH30 endo-β-1,4-xylanase XYN4	142.7	13.0	8 683.6	60.8
69944	GH31 α-xylosidase/α-glucosidase	23.7	0.0	415.1	17.5
60085	GH31 α-glucosidase	43.9	8.3	427.5	9.7
79960	GH47 α-1,2-mannosidase	271.5	3.6	5 769.6	21.2
120312	GH5 endo-β-1,4-glucanase EGL2/CEL5a	9.7	0.0	29 758.3	3053.1
49976	GH5 endo-β-1,4-glucanase EGL5/CEL45a	158.7	3.6	25 111.8	158.2
56996	GH5 β-mannanase MAN1	0.7	0.0	226.1	324.7
55319	GH54 α-L-arabinofuranosidase ABF3	118.4	4.7	10 592.8	89.5
70845	GH55 β-1,3-glucanase	237.4	60.4	2 030.4	8.6
72567	GH6 cellobiohydrolase CBH2/CEL6a	245.1	7.1	372 237.7	1518.9
76210	GH62 α-L-arabinofuranosidase ABF2	0.0	0.0	115.1	ND
72526	GH67 α-Glucuronidase GLR1	337.7	13.0	7 793.8	23.1
123989	GH7 cellobiohydrolase CBH1/CEL7a	985.1	7.1	1 255 793.1	1274.7
122081	GH7 endo-β-1,4-glucanase EGL1/CEL7b	142.7	1.2	180 819.3	1266.9
49081	GH74 xyloglucanase CEL74a	394.8	360.3	8 088.7	20.5
74198	GH92 α-1,2-mannosidase	44.6	24.9	897.0	20.1
79921	GH92 α-1,2-mannosidase	503.4	88.9	3 199.7	6.4
58802	GH95 α-L-fucosidase	37.6	8.3	494.2	13.1
5807	GH95 α-L-fucosidase	32.7	5.9	319.6	9.8
123992	Swollenin	595.3	55.7	34 626.7	58.2

a Ratio of induction by sophorose in the retransformant strain Δkap8 RT over *Δkap8*.

AA, accessory enzyme; CE, carbohydrate esterases; GH, glycosyl hydrolases; glyc, glycerol; RPKM, reads per kilobase of exon per million mapped sequence reads; soph, sophorose.

### The A. nidulans KAP8 *ortholog KapI is not involved in growth on cellulose or xylan*

**Figure 4:**
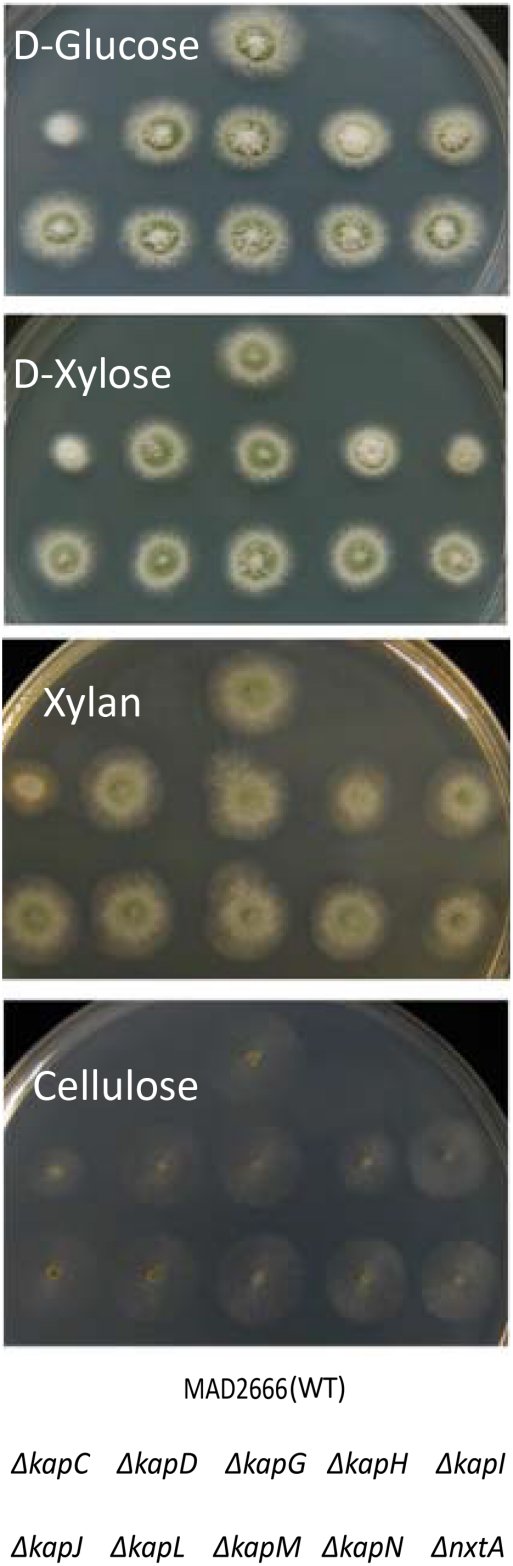
Growth of *A. nidulans* MAD2666 and mutant strains defective in selected *kap* genes (see Supporting Information Table S1) on D-glucose, the cellulose derivate CMC, xylan and D-xylose. Data from a single experiment are shown, but three replicates yielded consistent results. The order of strains and *kap* mutants for all carbon sources is shown in the sketch at the bottom of the figure.

Our findings that KAP8 regulates XYR1 nuclear import and consequently cellulase and hemicellulase formation in *T. reesei* prompted us to investigate whether this mechanism is also conserved in other fungi. We chose to test this in *A. nidulans* because its karyopherins have been studied in detail and mutants are available (Markina-Iñarrairaegui *et al*., 2011). In order to test whether the KAP8 ortholog KapI is involved in the function of the *A. nidulans* XYR1 ortholog XlnR, we cultivated Δ*kapI* and other *kap* mutant strains on D-glucose, D-xylose, birchwood xylan and carboxymethyl-cellulose, and monitored their growth. Induction of the enzymes required for catabolism of D-xylose, extracellular depolymerization of xylan and (in part) of cellulose has been shown to be dependent on XlnR function in *Aspergillus* spp. (Tsukagoshi *et al*., 2001; Tamayo *et al*., 2008). We thus expected that growth on these carbon sources would be reduced in the *A. nidulans* Δ*kapI* strain if KapI imported XlnR into the nucleus. As shown in Fig. 4, the *A. nidulans* Δ*kapI* strain grew equally well as the wild-type strain on D-glucose, xylan and cellulose, although a slightly reduced growth was observed on D-xylose. None of the other importin mutants showed any effect on the tested carbon sources. These data illustrate that KapI does not seem to have a major effect on XlnR nuclear import or that its function can be compensated by another karyopherin.

### Trichoderma reesei *KAP8 function is required for asexual – but not sexual – development*

**Figure 5:**
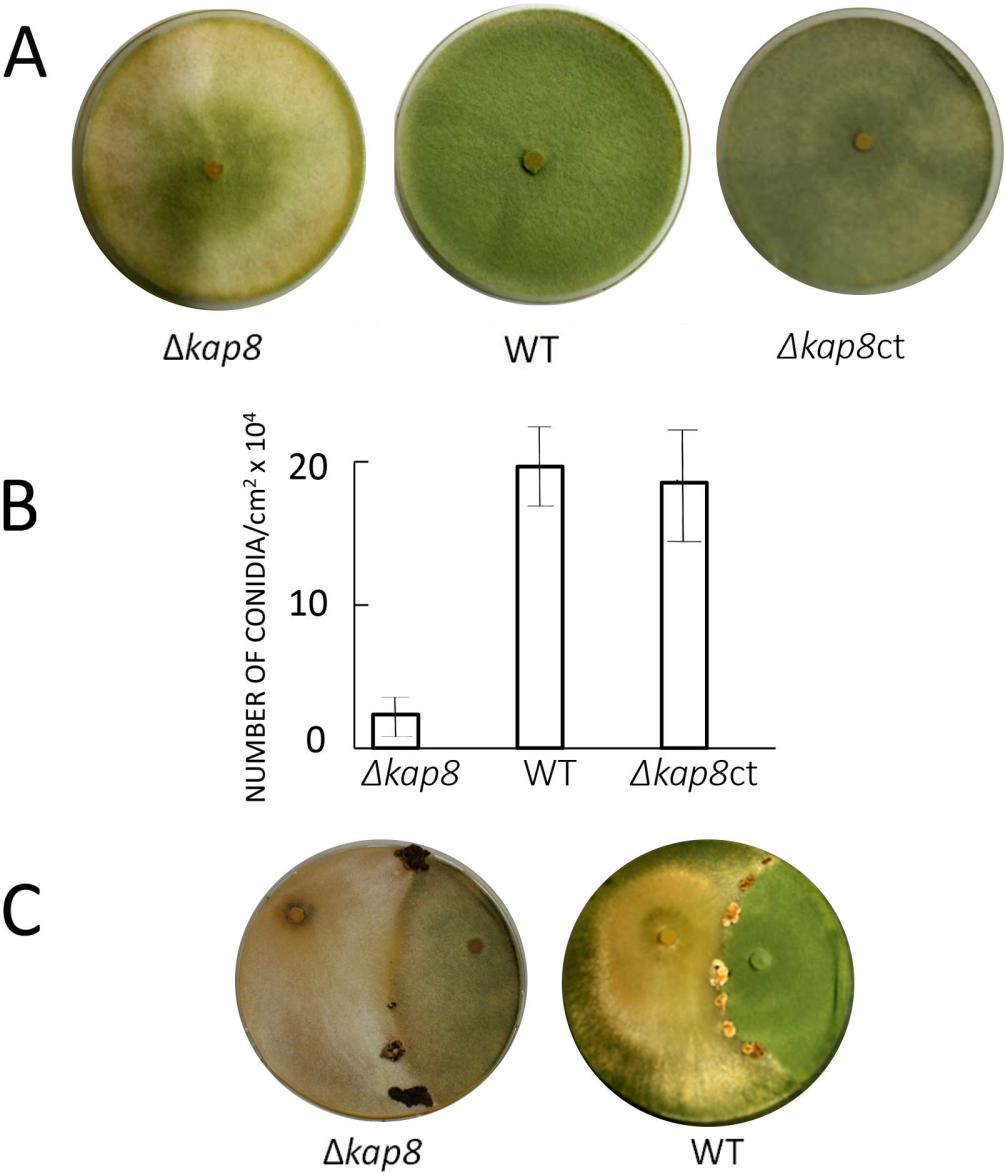
Effect of *kap8* knock out on asexual and sexual development in *T. reesei*:A. Phenotype of *Δkap8*, the parental strain and the complemented transformant (*Δkap8*ct) on PDA plates.B. Quantification of conidia harvested from these cultures. Vertical bars indicate standard deviations (*n* ≥ 3).C. fruiting body formation in confrontation with strain CBS999.79 (*MAT1-1*).

In *A. nidulans*, conidiation of the Δ*kapI* strain is reduced by two orders of magnitude compared with the isogenic wild-type strain (Etxebeste *et al*., 2009). In *T. reesei*, sporulation of the *Δkap8* strain was also reduced to < 10% of that of the parental strain (*n* = 4; Fig. 5A and B). In contrast to *A. nidulans*, however, we found no reduced hyphal extension rate in *T. reesei Δkap8* on D-glucose as a carbon source (0.14 vs. 0.11 cm h^−1^; SD ± 0.02 cm h^−1^). Also, no significant differences were detected with respect to hyphal branching frequency, measured as the average length of hyphae between any two branches in germlings and at the periphery of mature colonies. In addition, the *Δkap8* strain and its complemented transformant grew at a similar colony extension rate and with a similar biomass yield in liquid medium on D-glucose as carbon source (data not shown).

In order to test whether KAP8 would be involved in the nuclear import of a transcription factor essential for sexual recombination, we mated *T. reesei Δkap8 MAT1-2* with a corresponding *T. reesei MAT1-1* partner (see *Experimental procedures*). As shown in Fig. 5C, *T. reesei Δkap8* exhibited normal sexual development by producing fertile fruiting bodies in the same number and within the same time as the parent. We therefore conclude that KAP8 is not involved in nuclear transport of components essential for sexual development of *T. reesei*.

### Trichoderma reesei *KAP8 is involved in the general stress response*

**Figure 6:**
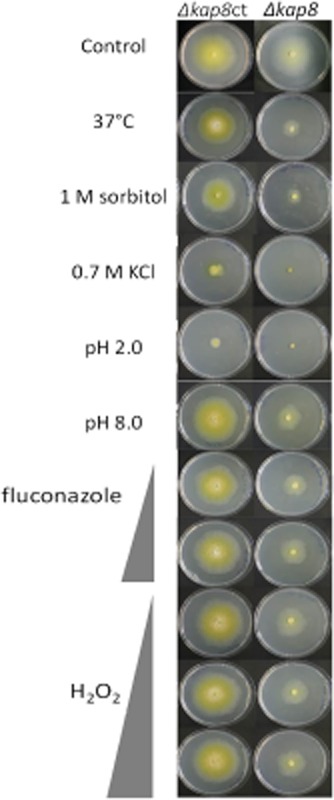
Growth of *T. reesei Δkap8* and the complemented transformant (*Δkap8*ct) on PDA in the presence of various stress-inducing agents: from top to bottom 1.5, 10 and 20 mM H_2_O_2_; from top to bottom 0.5 and 2 μg ml^−1^ fluconazole. Other concentrations/conditions are directly indicated. The plates shown are from a single experiments, but two further biological replicates yielded consistent results.

In *S. cerevisiae*, Kap121 is essential for the nuclear import of the oxidative stress regulator Yap1 (Isoyama *et al*., 2001) and the antibiotic efflux regulator Pdr1 (Delahodde *et al*., 2001; Caudle *et al*., 2011). We have therefore investigated whether the deletion of *T. reesei kap8* might have an effect on its response to stress. To this end, we cultivated *T. reesei Δkap8* and its complemented transformant under conditions known to elicit a stress response, such as high concentrations of sorbitol and KCl (osmotic and salt stress), extreme pH, growth-inhibiting temperature (37°C), fluconazole (azole toxicity) and H_2_O_2_ (oxidative stress). The results, shown in Fig. 6, document that indeed the *Δkap8* strain exhibits significantly decreased growth under all these conditions, but the effect was most severe under osmotic stress, at low pH (pH2) and at elevated temperature (37°C). In contrast to what is observed in *A. nidulans* (Etxebeste *et al*., 2009), none of these stress conditions rescued the sporulation deficiency of the *Δkap8* strain.

**Figure 7:**
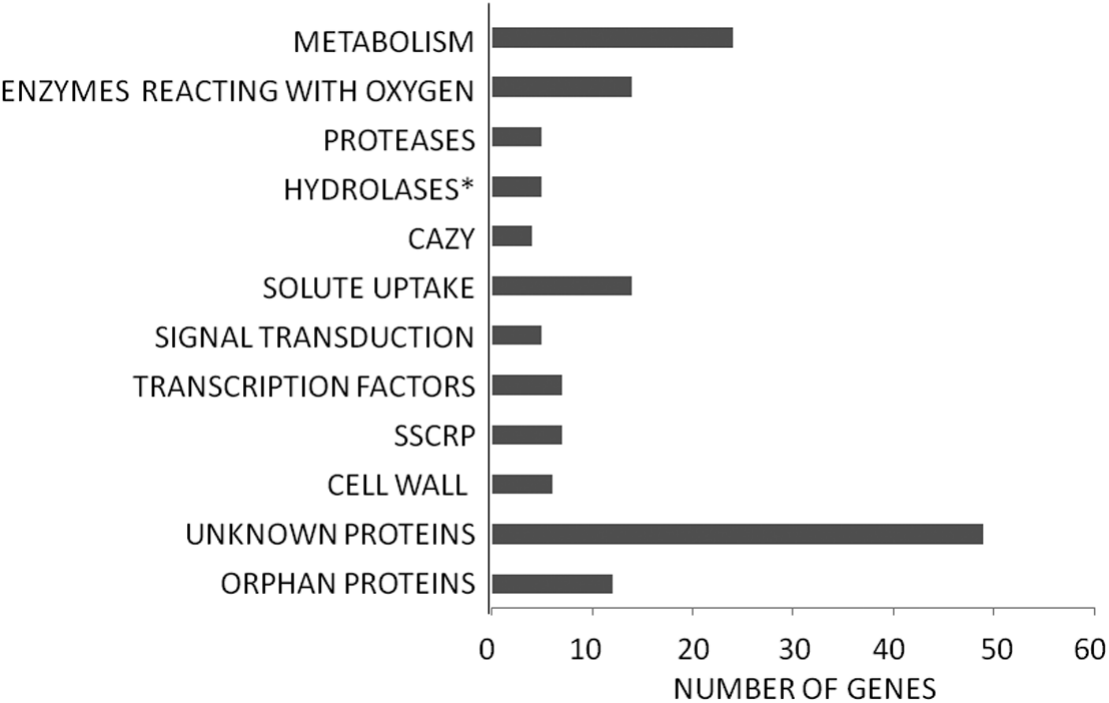
Inventory of gene groups that were not induced by sophorose but significantly down-regulated (at least > 5-fold; *P* < 0.05) in the *Δkap8* strain compared with the retransformant. The gene groups encounter 152 from 163 down-regulated genes (for full description, see Supporting Information Table S6). Specification of gene groups is as described in the legend to Fig. 3. New groups include HYDROLASES, extracellular lipases, esterases and amidases; SIGNAL TRANSDUCTION, involved in signal transduction pathways; and CELL WALL, proteins being components of the fungal cell wall, glycosyltransferases involved in their biosynthesis.

To potentially identify genes that could be causally related to this reduced stress resistance, we searched our transcriptomic data for genes whose expression was KAP8 dependent but not linked to cellulose induction. Thus, we looked for genes whose expression on glycerol was either similar or even higher as that on sophorose but strongly reduced (> 5-fold, *P* < 0.05) under both conditions in the *Δkap8* strain. One hundred sixty-three genes fulfilled this criterion (Supporting Information Table S6). Some of the major gene groups in this sample were the same as during induction with sophorose (i.e. genes encoding metabolic enzymes, MFS permeases and unknown proteins; Fig. 7). Among the other genes, however, a few examples are noteworthy, e.g. the two-component histidine kinase Trire2:70943; the aquaglyceroporin Trir2:81082; three PTH11-type G-protein coupled receptors Trire2: 82041, 69904 and 122795; the translation initiation regulator GCN20 (Trire2: 22839); several putative cell wall proteins (Trire2: 123659,123475; the *Trichoderma* cell wall protein QI74 Trire2:74282 (Rey *et al*., 1998); and five Zn(2)Cys(6) transcriptional activators of which the expression of two (Trire2: 112036, 112560) was absolutely dependent on KAP8 function.

## Discussion

In this paper, we provide evidence that the *T. reesei* ß-importin KAP8, an ortholog of *S. cerevisiae* Pse1/Kap121 and *A. nidulans* KapI, is essential for the nuclear import of the transcriptional regulator for cellulase and hemicellulase gene expression XYR1. Pse1 was first reported in yeast as an auxiliary import receptor of ribosomal protein L25, since the defective import of L25 observed in the *kap123*Δ strain was reversed by the overexpression of Pse1 (Schlenstedt *et al*., 1997), and subsequently shown to be an essential protein that regulates multiple cellular transcription factors such as Pho4 (responding to phosphate stress; Kaffman *et al*., 1998), Yap1 (oxidative stress response; Isoyama *et al*., 2001), Pdr1 (membrane biogenesis; Delahodde *et al*., 2001), the iron regulator Aft1 (Ueta *et al*., 2003), Ste12 (mating response; Leslie *et al*., 2002) and a regulatory protein for sporulation (Chaves and Blobel, 2001). In filamentous fungi, the function of the *A. nidulans* KapI appears to be nonessential (Etxebeste *et al*., 2009), and here, we could confirm this also for *T. reesei* ortholog KAP8. Although its role in the control of cellular processes such as conidiation and hyphal branching has been demonstrated in *A. nidulans*, the transcriptional activators whose transport would be performed by KapI, and consequently impact the above processes, have not yet been identified (Etxebeste *et al*., 2009). Interestingly, cellulase gene transcription occurs in *T. reesei* during conidiation in the absence of an inducer, which would link XYR1 to sporulation. However, XYR1 has been shown to play no regulatory role in asexual sporulation (Metz *et al*., 2011).

Transcriptomic analysis showed that a loss of function of KAP8 led to impairment of the induction of all cellulase and xylanase genes of *T. reesei*. This impairment has also been shown for several other genes for which the regulation by XYR1 has not yet been demonstrated, most notably including α-D-galactosidases, α-D-mannosidases and α-D-fucosidases. Their XYR1-dependent induction by the cellulase inducer sophorose implies that these enzymes belong to the standard repertoire of *T. reesei* when faced with a lignocellulosic substrate, and support previous interpretations (Ivanova *et al*., 2013) that *T. reesei* is also strongly active on hemicellulose side chains. Häkkinen *et al*. (2012) have recently described the sophorose-inducible CAZome of *T. reesei* and listed 56 genes. Although we detected almost the same number (55), only 30 and 31 genes, respectively, were identical between the two studies (Supporting Information Table S7). We assume that our more stringent criteria for induction and the early time point (3 h) may be responsible for this difference, as well as the fact that RNAseq was used in the present work whereas (Häkkinen *et al*., 2012) used microarrays.

We also identified four transcription factors that were induced by sophorose and dependent on the function of KAP8. Interestingly, none of them has been found in a screening of *T. reesei* transcription factors that correlate with cellulose formation (Häkkinen *et al*., 2014). Their relationship to cellulase and hemicellulase gene expression, if any, needs to be assessed, but this was beyond the scope of the present paper. The already known regulators of cellulose and hemicellulase gene expression in *T. reesei* (i.e. XYR1, ACE3, and CLR2) were all significantly induced by sophorose, and their expression was reduced in the *Δkap8* strain. However, all of them exhibited a significant residual level of expression. Therefore, they all failed to pass our criteria for regulation by KAP8.

It is intriguing to note in this context that KAP8 and also the nuclear transport factor 2 (Trire2:22294; an essential component for the small GTPase Ran which assists in the nuclear export of ß-importins) and ataxin-7 (Trire2:112346; which ‘gates’ proteins to the nucleopore complex) have been shown to have become mutated during generation of the *T. reesei* cellulase hyperproducer mutant RUT C30 from its parent NG14 (Le Crom *et al*., 2009). However, despite of the importance of *kap8* for cellulase and hemicellulase formation as we have shown here, the mutation found in RUT C30 unlikely contributes to the increased cellulase production in this strain because other mutations were meanwhile found that make up for most of the differences in productivity between RUT C30 and its parent strain (C. Ivanova and B. Seiboth, unpubl. data).

Having identified KAP8 as the importin that is responsible for nuclear import of XYR1 and consequently cellulase and hemicellulase gene expression in *T. reesei*, we decided to look for similar functional conservation in other fungi. We chose *A. nidulans* for this purpose because knockout strains in all importins are available for this species and because the role of XlnR in growth on xylan and xylanase gene expression has been documented (Tamayo *et al*., 2008). However, we found that the *A. nidulans kapI* knockout strain grew as well on xylan as its parent thus arguing against a role of KapI in XlnR function. There was also no difference between *ΔkapI* and its parent with respect to growth on cellulose, but we must note that a role of XlnR in regulation of cellulase gene expression in *Aspergilli* is not clear (De Souza *et al*., 2013). As noted above, regulation of cellulose gene expression in *A. nidulans* and *N. crassa* is mainly performed by CLR-2/ClrB (Coradetti *et al*., 2012), and thus, the role of XYR1 in cellulase gene transcription in *T. reesei* is so far unique among fungi. The ability of ΔkapI mutants to grow well on cellulose indicates that KapI is either not or not exclusively involved in nuclear transport of ClrB in *A. nidulans*, and this fits also to our discussion of its *T. reesei* ortholog CLR2.

This defect in conidiation of the *A. nidulans ΔkapI* strain is suppressed under abiotic stress, whereas this was not the case in *T. reesei*. In accordance with *S. cerevisiae* (Kaffman *et al*., 1998; Isoyama *et al*., 2001), *T. reesei* KAP8 is necessary for a full stress response, and the *Δkap8* strain is particularly sensitive against low pH and osmotic stress. Our transcriptomic data revealed two genes that were strongly down-regulated in the *Δkap8* strain and that could therefore be involved in *T. reesei* osmotic stress, i.e. the class VI histidine kinase SLN1 (Trir2:70943) and an aquaglyceroporin (Trir2:81082). In *S. cerevisiae*, Sln1 is involved in the high-osmolarity stress response by transmitting the osmolarity signal through the Sln1-Ypd1-Ssk1 two-component system and the Ssk2/Ssk22-Pbs2-Hog1 MAP kinase cascade (Hohmann, 2002). Whether Sln1-orthologs would function in the same way in Pezizomycota is unclear; however, in *Aspergillus spp.*, targeted deletion of the *Sln1* ortholog produced no stress-impaired phenotype (Du *et al*., 2006; Furukawa *et al*., 2008), whereas in the pyrenomycete *Magnaporthe oryzae*, which is evolutionary closer to *Trichoderma* (Wang *et al*., 2009), deletion of the Sln1 ortholog MoSLN1 significantly affected fungal growth and morphology on different media, and resulted in impaired resistance to oxidative and osmotic stress (Zhang *et al*., 2010). With regard to the aquaglyceroporin (Trir2:81082), *S. cerevisiae* maintains the osmotic equilibrium und osmotic stress by producing and retaining high concentrations of glycerol as a compatible solute, whose intracellular concentration is to a large extent determined by the regulated activity of aquaglyceroporins. A similar mechanism may occur in *T. reesei*, yet it still requires testing.

We have identified a major player in the import of XYR1 and cellulose and hemicellulose gene expression. However, we have also shown that the expression of XYR1 – despite being triggered by cellulase inducers – is only partially impaired by loss of function of *kap8*. This implies that *xyr1* induction is only partially due to XYR1-dependent autoregulation (Lichius *et al*., 2014) and thus requires other as yet unknown transcription factors whose nuclear recruitment is KAP8 independent. Identification of this factor may also open new avenues for improvement of cellulose production in *T. reesei*.

## Experimental procedures

### Strains and culture conditions

The *Trichoderma reesei* strain QM9414 (ATCC 26921) and recombinant mutants derived from it were used throughout this work. Strain propagation, transformant selection and purification were performed on potato dextrose agar (PDA). For experimentation, strains were grown in Mandels-Andreotti medium (Mandels and Andreotti, 1978) using glucose, lactose or cellulose [in the form of carboxymethylcellulose (CMC)] as sole carbon sources at final concentrations of up to 1% (w/v) as indicated. Induction by sophorose was performed by replacing fungal biomass from 24 h 1% (w/v) glycerol precultures into fresh medium containing 1.4 mM sophorose as only carbon source as described by Sternberg and Mandels (1979). All strains are maintained as 50% (v/v) glycerol stocks at −80°C at TUCIM (http://www.vt.tuwien.ac.at/tucim/). Table 3 lists all *T. reesei* strains used and produced in this study.

**Table 3:** *Trichoderma reesei* strains and genotypes used in this work

Strain	Genotype	Reference
*T. reesei* QM9414	*mat1-2*	Mandels and Andreotti (1978)
*T. reesei* CBS 999.79	*mat1-1*	Seidl *et al*. (2009)
*T. reesei Δtku70*	*Δtku70 pyr4- mat 1-2.*	Guangtao *et al*. (2009)
*T. reesei gfp-xyr1*	*Δtku70 pyr4- gfp-xyr1::nat1 mat 1-2.*	Lichius *et al*. (2014)
*T. reesei Δxyr1*	*Δxyr1 amdS- mat 1-2.*	Stricker *et al*. (2006)
*T. reesei Δkap8*	*Δtku70 Δkap8::pyr4 pyr4- mat 1-2.*	This study
*T. reesei Δkap8ct*	*Δtku70 Δkap8::pyr4 pyr4- kap8::nat1 mat 1-2.*	This study
*T. reesei Δkap8 gfp-xyr1*	*Δtku70 Δkap8::pyr4 pyr4- gfp-xyr1::hph mat 1-2.*	This study
*T. reesei Δkap8 ct gfp-xyr1*	*Δtku70 Δkap8::pyr4 pyr4- kap8::nat1 gfp-xyr1::hph mat 1-2.*	This study
*T. reesei Δkap1*	*Δtku70 Δkap1::pyr4 pyr4- mat 1-2 + Δtku70 pyr4- mat 1-2*	This study
*T. reesei Δkap2*	*Δtku70 Δkap2::pyr4 pyr4- mat 1-2 + Δtku70 pyr4- mat 1-2*	This study
*T. reesei Δkap4*	*Δtku70 Δkap4::pyr4 pyr4- mat 1-2.*	This study
*T. reesei Δkap5*	*Δtku70 Δkap5::pyr4 pyr4- mat 1-2 + Δtku70 pyr4- mat 1-2*	This study
*T. reesei Δkap6*	*Δtku70 Δkap6::pyr4 pyr4- mat 1-2.*	This study
*T. reesei Δkap7*	*Δtku70 Δkap7::pyr4 pyr4- mat 1-2.*	This study
*T. reesei Δkap9*	*Δtku70 Δkap9::pyr4 pyr4- mat 1-2.*	This study
*T. reesei Δkap10*	*Δtku70 Δkap10::pyr4 pyr4- mat 1-2.*	This study

The *A. nidulans* strains used in this study are given in Supporting Information Table 3. The *pyrG89* mutant strain TN02A3 served as parental strain for the importin knockout mutants. A *pyrG* complemented transformant of TN02A3 (MAD2666) served as a comparison for the growth experiments. All *A. nidulans* mutants were produced in the lab of Eduardo Espeso, CIB, Madrid, Spain (Markina-Iñarrairaegui *et al*., 2011), and propagated on complete medium plates at 37°C. The wild-type and the mutant strains were transferred to minimal medium plates containing either 1% glucose or 1% CMC as carbon source. Biotin, ammonium tartrate and pyridoxine were added to the medium.

*Escherichia coli* strains JM109 (Promega, Madison, Wisconsin) or Stellar® (#636763, Takara Bio Europe/Clontech, Saint-Germain-en-Laye, France) were used for plasmid construction and amplification using standard molecular cloning techniques (Sambrook and Russell, 2001).

### Nucleic acid isolation and hybridization

Fungal biomass was harvested by vacuum filtration, washed with precooled, distilled and sterile water, and shock frozen and ground in liquid nitrogen. For extraction of genomic DNA, plasmid DNA and RNA, purification kits (Wizard Genomic DNA Purification Kit, PureYield Plasmid Midiprep System and RNeasy plant kit, respectively, all from Promega) were used according to the manufacturer's specifications. Standard methods were used for electrophoresis, blotting and hybridization of nucleic acids (Sambrook and Russell, 2001).

### *Construction of* T. reesei *recombinant strains*

To study the function of importins, we constructed *T. reesei* strains with individual deletions in all encoded importin genes. *T. reesei* strain *Δtku70* (Guangtao *et al*., 2009) was used as a recipient for all targeted gene deletions. Specifically, the coding region of the individual importin genes was replaced by the *T. reesei pyr4* (orotidine-5′-phosphate decarboxylase-encoding) gene (Gruber *et al*., 1990), flanked by 5′ and 3′ noncoding sequences of the respective importins. To this end, 1.2 kb of the up and downstream noncoding regions of the respective importins were amplified from genomic DNA of *T. reesei Δtku70* with the primer pair series 5f+5r and 3f+3r. The 5′ and 3′ ends of 5r and 3r bear sequences homologous to the *pyr4* gene, whereas the primer series 5f and 3r have 5′ and 3′ ends complementary to the pRS426 yeast vector. The nucleotide sequences of all primer pairs used are given in Supporting Information Table S2. The thus resulting PCR fragments of 5′ and 3′ pRS426 and the 2.7 kb fragment of the *pyr4* gene were subsequently recombined into the *Xho*I/*Eco*RI linearized vector backbone of pRS426 using the endogenous yeast homologous recombination system, and the resulting gene replacement cassette was electro-transformed into *T. reesei* (Schuster *et al*., 2012).

Verification of the putative deletion mutants was performed by PCR: integration of the deletion construct into genomic DNA was shown using the primers 5p200 (which binds in 5′-noncoding region of the respective importin that is not present in the deletion vector) and pyr4R (which binds inside the selection marker *pyr4*). In addition, the absence of the native importin gene was shown by PCR with primers 5p200 (*vide supra*) or GeneX-ORF-F (which binds in the 5′-quarter of the importin coding region) and x-1000-R (which binds in the 3′-quarter of the importin coding region).

To verify that the effects observed with the *Δkap8* strain were indeed specific for this gene, we also investigated them in a *Δkap8* strain that had been transformed with the native *kap8* gene by employing nourseothricin as second selection marker (Kück and Hoff, 2006; Lichius, 2010). Strains in which *kap8* had been again reintegrated ectopically (termed *Δkapct for complemented transformant* throughout) were identified by PCR for the presence of *kap8* as just described. To express an N-terminal GFP-fusion protein of XYR1 in *T. reesei Δkap8* and its Δ*kap8ct*, respective strains were transformed with a *gfp-xyr1* gene replacement cassette conferring resistance against hygromycin (Lichius *et al*., 2014), and positively genotyped by PCR accordingly.

### RNA-seq library preparation and analysis

Library preparation and Illumina sequencing were performed at the École Normale Supérieure Genomic Platform (Paris, France). Messenger (polyA+) RNAs were purified from 800 ng of total RNA using oligo(dT). Libraries were prepared using the stranded RNA-Seq library preparation TruSeq RNA Sample Prep Kits (Illumina). Libraries were multiplexed by four on three single flow cell lanes and subjected to 50 bp paired-end read sequencing on a HiSeq 2000 device. A mean of 47 ± 6 million passing illumina quality filter reads was obtained for each of the 12 samples. Transcriptomic analysis was performed as three experimental replicates from which mean values were calculated and presented. RNA-Seq data analysis was done using the Eoulsan software version 1.2.2 (Jourdren *et al*., 2012). Before mapping, poly N read tails were trimmed, reads ≤ 40 bases were removed, and reads with quality mean ≤ 30 were discarded. Reads were then aligned against the *T. reesei* reference genome (http://genome.jgi-psf.org/Trire2/Trire2.home.html) using Bowtie mapper (version 0.12.7) using the ‘-n 2 -l 34 -e 70 -k 2 --best’ parameters. Only one alignment was kept in a given locus for each read, and read alignments matching on more than one locus were removed. To compute gene expression, the *T. reesei* genome annotation was used Gene expression was computed by counting all overlapping regions between alignments and referenced exons. To quantify the gene expression level, the relative transcript abundance was measured in reads per kilobase of exon per million mapped sequence reads (RPKM; (Mortazavi *et al*., 2008)). The log2 ratios of the RPKM values were used to identify differentially expressed genes. To keep only the most differentially expressed genes, thresholds of four (sophorose vs. glycerol in the retransformant) and five (retransformant vs. *Δkap8* on sophorose) for log ratio were used. Read numbers < 100 were considered as (almost) absence of transcription and not chosen for the evaluation. Genes were identified by the aid of a completely manually annotated *T. reesei* genome database proprietary to C.P.K.

The RNA-Seq gene expression data and raw fastq files are available at the GEO repository (http://www.ncbi.nlm.nih.gov/geo/) under accession number GSE59600.

### Analysis of sexual and asexual development

To test the effect of importin gene deletion on sexual reproduction, *T. reesei MAT1-2* parental and mutant strains were confronted with the compatible *MAT1-1* mating partner CBS999.79 as described previously (Seidl *et al*., 2009). All crosses in which fruiting bodies were formed were visually inspected until the maturation stage was achieved and ascospores were dispersed. Monoascospore cultures were isolated by dispersing the solution with a cotton swab on multiple PDA plates. After overnight incubation, several single germinated spores were selected with on a stereomicroscope, transferred to a new PDA plate and cultivated at 28°C.

To test for photo-dependent conidiation, PDA plates were centrally inoculated with one 5 mm diameter mycelial plug taken from the edge of a 3-day-old colony and incubated for eight days at 28°C in either complete darkness or cycles of 12 h illumination/12 h darkness. Three biological replicates were prepared for each condition. Conidia from each plate were then harvested in equal volumes of physiological salt solution (0.1%, w/v, Tween 80 and 0.8% w/v NaCl) by gently rubbing the surface of the mycelium with a sterile Drygalski spatula. The crude spore suspension was then filtered through glass wool to remove mycelial fragments, and conidia were sedimented by centrifugation (5000 × *g*, 10 min). Finally, the spore pellet was resuspended in 2.5 g l^−1^ phytagel (Phytagel^TM,^ SIGMA, Steinheim, Germany) and well mixed, and the transmission at 590 nm in a Biolog standard turbidimeter was measured. The number of total conidia was calculated using a calibration curve previously prepared with *T. reesei* QM9414 conidia.

### Enzymatic assays and determination of fungal dry weight

Cellulase enzyme activities were determined using 1% (w/v) CMC and p-nitrophenyl-ß-D-lactobioside (Bailey and Tahtiharju, 2003). Total protein in the culture supernatant was determined by the method of Bradford (Bradford, 1976). Fungal dry weight was determined by filtering an aliquot of the culture through glass sinter funnels (porosity G1), washing with tap water and drying at 80°C to constant weight.

### Analysis of gene expression by quantitative RT-PCR

This was performed as described recently (Lichius *et al*., 2014). Briefly, Dnase-treated (DNase I, RNase free; Fermentas) RNA (5 μg) was reverse transcribed with the RevertAid™ First Strand cDNA Kit (Fermentas) according to the manufacturer's protocol with a combination of oligo-dT and random hexamer primers, and all qPCR assays were performed on a Bio-Rad (Hercules, CA) iCycler IQ. Relative gene expression ratios were calculated using REST^©^ Software (Pfaffl *et al*., 2002). All samples were analyzed in at least two independent experiments with three replicates in each run.

The significance of differences in gene expression between different knockout mutants and the remaining strains at a given time point was evaluated by the Student's *t*-test (http://studentsttest.com/), assuming unequal variance of groups. To this end, a group consisting of the replicate values of a given knockout strain was compared against a group containing all replicas of all the other knockout strains.

### Monitoring of XYR1 nuclear transport

Expression and subcellular localization of GFP-labeled fusion proteins was investigated by fluorescence microscopy and image analysis as described recently (Lichius *et al*., 2014). Live cell imaging was performed using a Nikon C1 confocal laser scanning unit mounted on a Nikon Eclipse TE2000-E inverted microscope base (Nikon GmbH, Vienna, Austria). GFP-labeled proteins were excited with the 488 nm laser line of an argon ion laser, and emitted fluorescence light separated by a Nikon MHX40500b/C100332 filter cube was detected with a photo-multiplier tube within the range of 500–530 nm. A Nikon Plan Apo VC 60× /1.2 water immersion objective lens was used, and laser intensity and laser dwell time during image acquisition were kept to a minimum to reduce photobleaching and phototoxic effects. Brightfield images were captured simultaneously with a Nikon C1-TD transmitted light detector mounted behind the condenser turret. Images were recorded with a maximum resolution of 1024 × 1024 pixels and saved as TIFF. HOECHST dye #34580 (*life* technologies, Invitrogen, cat# H21486) was used at a final concentration of 3.6 μM to counterstain nuclei. HOECHST fluorescence was detected in the range of 430–470 nm, after excitation of the dye with 405 nm of a HeNe laser and separation through a Nikon MHX40500a filter cube. This setup, unfortunately, was not capable of fully eliminating bleed-through of strong HOECHST signals into the GFP channel even when using sequential scanning.
